# No Additional Effects of Sequential Facilitatory Cerebral and Cerebellar rTMS in Subacute Stroke Patients

**DOI:** 10.3390/jpm14070687

**Published:** 2024-06-26

**Authors:** Ho Seok Lee, Sungwon Kim, Heegoo Kim, Seung-min Baik, Dae Hyun Kim, Won Hyuk Chang

**Affiliations:** 1 Department of Physical and Rehabilitation Medicine, Center for Prevention and Rehabilitation, Heart Vascular and Stroke Institute, Samsung Medical Center, Sungkyunkwan University School of Medicine, Seoul 06351, Republic of Korea; 2 Department of Health Sciences and Technology, Department of Medical Device Management & Research, Department of Digital Health, SAIHST, Sungkyunkwan University, Seoul 06355, Republic of Korea

**Keywords:** repetitive transcranial magnetic stimulation, stroke, motor learning, cerebellum, rehabilitation

## Abstract

The aim of this study was to investigate the additional effects of cerebellar rTMS on the motor recovery of facilitatory rTMS over affected primary motor cortex (M1) in subacute stroke patients. Twenty-eight subacute stroke patients were recruited in this single-blind, randomized, controlled trial. The Cr-Cbll group received Cr-Cbll rTMS stimulation consisting of high-frequency rTMS over affected M1 (10 min), motor training (10 min), and high-frequency rTMS over contralesional Cbll (10 min). The Cr-sham group received sham rTMS instead of high-frequency rTMS over the cerebellum. Ten daily sessions were performed for 2 weeks. A Fugl-Meyer Assessment (FMA) was measured before (T0), immediately after (T1), and 2 months after the intervention (T2). A total of 20 participants (10 in the Cr-Cbll group and 10 in the Cr-sham group) completed the intervention. There was no significant difference in clinical characteristics between the two groups at T0. FMA was significantly improved after the intervention in both Cr-Cbll and Cr-sham groups (*p* < 0.05). However, there was no significant interaction in FMA between time and group. In conclusion, these results could not demonstrate that rTMS over the contralesional cerebellum has additional effects to facilitatory rTMS over the affected M1 for improving motor function in subacute stroke patients.

## 1. Introduction

Motor impairment is one of the most common complications in stroke patients [1]. In particular, reduced upper extremity function significantly affects the daily life of stroke patients and increases the burden of on the patients and their supporting families [2]. Novel strategies to improve the recovery of upper extremity function are needed, as specific rehabilitation interventions available in routine clinical practice are still insufficient for most patients [3]. As a safe and effective non-invasive method of brain stimulation, repetitive transcranial magnetic stimulation (rTMS) is a relatively new approach to stroke rehabilitation [4]. The most common rTMS strategies for improving motor function in stroke patients are facilitation of the affected primary motor cortex (M1) and inhibition of the unaffected M1 [4]. Facilitatory high-frequency rTMS of the affected primary motor cortex has been shown to have positive long-term effects on motor recovery in subacute stroke patients [5,6,7]. However, the clinical effect size of this rTMS strategy for improving motor function in stroke patients was relatively small [8].

Previous studies showed that the cerebellum has strong anatomic and functional connections to M1 via cerebellar-thalamus-M1 circuits [9,10]. Deep cerebellar nuclei form an excitatory synaptic connection with the motor cortex through the ventral thalamus [10]. In addition, the cerebellum is reciprocally connected to the basal ganglia, suggesting that these two subcortical structures are part of a densely interconnected network [11] and abnormal activity in basal ganglia circuits with the cerebral cortex and with the cerebellum may contribute to motor deficits associated with several neurological disorders [12]. The structural connectivity between the cerebellum and the motor cortex plays a role in functional reorganization, residual motor functions, and recovery after stroke [13,14]. Based on these studies, the cerebellum has recently been reported as a new target for rTMS in stroke patients [10,15,16]. These studies with cerebellar rTMS have primarily focused on improving balance and lower extremity motor function in stroke patients [10,16]. In addition, although several studies have reported the effects of cerebellar rTMS in stroke patients [10,15,16,17], there is a lack of research on the effects of sequential M1 and cerebellar rTMS. The mechanism by which rTMS improves upper limb motor function in subacute stroke patients is that it can efficiently modulate the motor learning process [18]. Facilitatory high-frequency rTMS of M1 has been proposed on the basis that M1 is important for initiating the development of long-term motor memory [19]. Previous studies have reported that the cerebellum is also involved in motor learning in conjunction with other critical areas, including M1 [20,21,22,23]. Several previous functional neuroimaging studies suggested that the cerebellum might be one of the main regions for the motor learning process [22,24,25]. Serial functional magnetic resonance imaging (fMRI) during motor learning tasks showed an initial activation and subsequent decrease in activity in the cerebellar cortex, as well as a subsequent increase in activity in the cerebellum [22,24,25]. Especially, the activity of the cerebellum correlated with the degree of savings at transfer of motor learning [24]. These previous functional neuroimaging studies suggest that the cerebellum may be a novel target for rTMS, as the cerebellum may play an important role in both early motor learning and the consolidation process of motor learning. According to these studies, the cerebellum may be one of the targets of rTMS for improving motor function in stroke patients, but evidence is limited.

The hypothesis of this study was that the sequential application of facilitatory rTMS to the cerebellum could enhance the effects of facilitatory rTMS over the affected M1 in patients with subacute stroke. The aim of this study was to investigate the additional effects of cerebellar rTMS on conventional facilitatory rTMS for motor recovery in subacute stroke patients.

## 2. Materials and Methods

### 2.1. Participants

Participants with subacute hemiplegic stroke were enrolled in an inpatient rehabilitation unit. Inclusion criteria were as follows: (1) hemiplegic cerebral stroke, (2) post-onset duration of greater than 7 days and less than 1 month, (3) moderate-to-severe motor impairment (Fugl-Meyer Assessment (FMA) score < 85), (4) response to TMS-induced MEPs of affected M1, (5) cognitive and language functions to perform more than one level of command, and (6) age of 19 years or older. Exclusion criteria were as follows: (1) contraindications to rTMS (history of seizures, presence of intracranial metallic implants), (2) progressive or unstable stroke defined as less than 4 on the National Institutes of Health Stroke Scale within 48 h, (3) pre-existing and active major neurological or major psychiatric illness by medical chart review, and (4) pregnant or lactating women.

The methods were approved by the Samsung Medical Center Institutional Review Board (2020-06-068), and this study adhered to the 2013 WMA Declaration of Helsinki. In addition, this study was conducted with the informed and written consent of each participant. The study protocol has been registered on ClinicalTrials.gov (NCT04570774).

### 2.2. Experimental Design

This study was a randomized, parallel, single-blinded, and sham-controlled trial. Participants were randomized into two groups: the cerebrum–cerebellum group (Cr-Cbll) or the cerebrum sham group (Cr-sham). Randomization was performed using an online random allocation tool (www.randomization.com accessed on 17 March 2019) by an investigator who had no contact with the participants. The participant allocation ratio for each group was 1:1. In this clinical trial, the assessment was performed by a blind observer who was unaware of the participant group. For the intervention, the Cr-Cbll group received Cr-Cbll rTMS and the Cr-sham group received Cr-sham rTMS. Motor function assessments were performed before (T0), immediately after (T1), and 2 months after the intervention (T2). During the intervention, participants in both groups received the same amount of conventional inpatient stroke rehabilitation.

### 2.3. Determination of MEPs Response and Stimulation Location

Transcranial magnetic stimulation-induced motor evoked potentials (TMS-induced MEPs) were assessed by single magnetic stimulations at 120% of resting motor threshold (rMT) over the affected M1 using a 70 mm figure-of-eight coil. Subjects were comfortably seated in an armchair with their eyes open during the experiments. A Synergy electromyography/evoked potential system (Medelec Co., Ltd., Kingswood, Bristol, UK) was used to record and monitor the activity of the contralateral first dorsal interosseus (FDI) muscle. Single-pulse TMS was applied over the affected M1 using a Magstim Rapid2^®^ stimulator (Magstim Co., Ltd., Spring Gardens, Whitland, Carmarthenshire, Wales, UK) equipped with a 70 mm figure-of-eight coil. The coil was held tangentially to the scalp with the handle pointing posteriorly and laterally at 45° from the mid-sagittal line. For TMS, the optimal position (“hot spot”) was defined as the location where TMS elicited MEPs of maximum peak-to-peak amplitude in the contralateral FDI muscle. rMT was defined as the lowest stimulus intensity capable of eliciting MEPs of at least 50 µV peak-to-peak amplitude on five out of ten consecutive trials. Five sweeps of MEPs at 120% of rMT were collected [26].

### 2.4. rTMS Intervention

**Figure 1:**
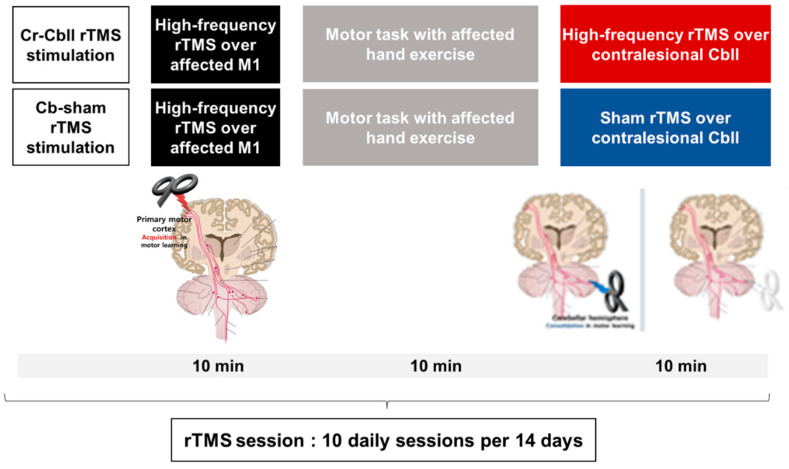
Cerebral and cerebellar repetitive transcranial magnetic stimulation protocol. rTMS: repetitive transcranial magnetic stimulation; Cr: cerebrum; Cbll: cerebellum; M1: primary motor cortex.

Ten daily intervention sessions were performed for two weeks in all participants. rTMS was delivered on the scalp over the affected M1 in accordance with safety recommendations [27] using a figure-of-eight coil connected to a Magstim Rapid^®^ stimulator with two booster modules (Magstim Co., Ltd., Spring Gardens, Whitland, Carmarthenshire, Wales, UK). The Cr-Cbll rTMS consisted of three steps, starting with high-frequency rTMS over the affected M1 for 10 min with 20 trains at 10 Hz for 5 s at 90% of the rMT and a 25 s intertrain interval (A total of 1000 pulses) [28]. This was followed by a motor task of the affected hand for 10 min. The motor task utilized block movements to facilitate the training of active grasping and releasing, thereby enhancing fine motor skills. Finally, high-frequency rTMS was applied over the contralesional cerebellum for 10 min. Cr-sham rTMS was identical to the first two steps but included sham rTMS using a sham coil over the contralesional cerebellum instead of high-frequency rTMS (Figure 1). High-frequency rTMS over the contralesional cerebellum consisted of 20 cycles, and each cycle consisted of 50 pulses at 10 Hz for 5 s and 25 s of rest at an intensity of 100% rMT of unaffected M1, for a total of 1000 pulses delivered. The stimulation site was 3 cm lateral to the palpated external occipital protuberance, and the coil was positioned tangential to the scalp with the handle pointing superiorly [28].

### 2.5. Baseline Characteristics and Outcome Measures

Participants’ medical records were collected for age, sex, stroke type, side of stroke, and duration after onset. Results regarding motor function domain of the Fugl-Meyer Assessment as the quantitative evaluative instrument for measuring sensorimotor stroke recovery [29] and the Box and Block Test (BBT) to measure unilateral gross manual dexterity [30] were obtained for all participants. Total, upper extremity, and lower extremity scores of the FMA (FMA-T, FMA-UE, and FMA-LE) were assessed separately. All assessments were performed by the same licensed occupational therapist who was blinded to the grouping of each participant. Throughout the intervention period, we planned to record all symptoms potentially related to the intervention, including seizures, headaches, neck discomfort, paresthesia, changes in hearing or vision, and syncope.

### 2.6. Statistical Analysis

The initial study design included total 38 participants, 19 in each group. However, the following additional modifications were made to the statistical analysis to validate it because we were unable to recruit enough participants during the limited study period. The primary outcome of this study was the difference in FMA-UE (ranging from 0 to 66) from T0 to T1. To estimate the sample size required for the study, we used Lehr’s formula [31]. We set the desired significance level (α) at 0.05 and the desired power (1-β) at 0.90. We determined the clinically important differences of the primary outcome to be 12.40 based on a previous study [32]. We estimated the standard deviation to be 8.30 and the follow-up rate to be 70.0%, which were taken from our previous study with participants with similar characteristics [33]. This process led us to conclude that we should recruit more than 28 participants for our study, 14 for each group.

The full analysis set was defined as the population of participants who underwent functional assessments at least at baseline (T0) and immediately after the intervention (T1). Missing data were imputed using the last observation carried forward (LOCF) method. To compare baseline demographic and clinical characteristics between the two groups, an independent *t*-test was used for continuous variables based on the assumption of normal distribution, confirmed by the Shapiro–Wilk test. All scores were found to be normally distributed (*p* > 0.05 by the Shapiro–Wilk normality test). Repeated measures analysis of variance (RM-ANOVA) was used to perform the analysis between groups and to test for the presence of an interaction between group and time (from T0 to T2). Post-hoc analyses were performed using paired *t*-test for within-group analysis and independent *t*-test for between-group analysis. Bonferroni correction was used to correct for multiple comparisons. Statistical significance was defined as *p*-value < 0.05.

## 3. Results

**Figure 2:**
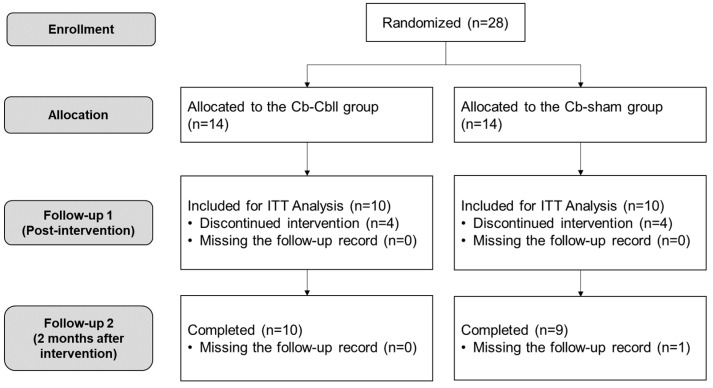
Flowchart of this study. ITT: intention to treat.

A total of 28 participants were enrolled in this study and randomized into two groups: 14 in the Cr-Cbll group and 14 in the Cr-sham group. Eight patients (four in the Cr-Cbll group and four in the Cr-sham group) withdrew during the intervention for personal reasons such as COVID-19 infection, a lack of time, or early discharge unrelated to the intervention, and one patient in the Cr-sham group missed the last follow-up visit. The intervention was well tolerated by all participants and no adverse effects were reported in 20 participants. Finally, 20 participants (10 in the Cr-Cbll group and 10 in the Cr-sham group) completed the intervention for 2 weeks and were included in the intention-to-treat analysis at T1 (Figure 2).

There were no significant differences in demographic and clinical characteristics between the two groups. There was no significant difference in motor function between the two groups at T0 (Table 1).

### 3.1. Changes in Fugl-Meyer Assessment

**Figure 3:**
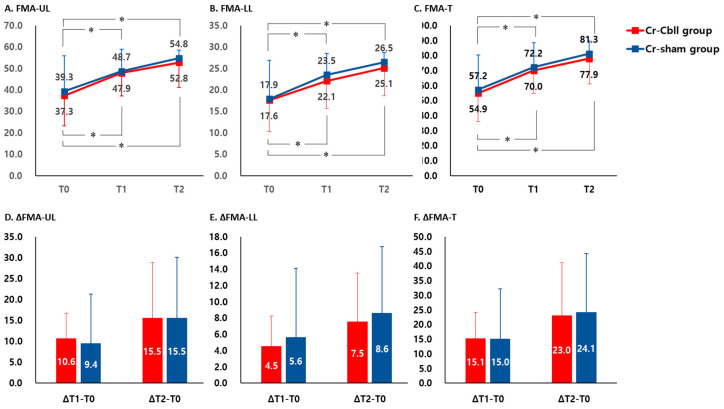
Changes in Fugl-Meyer Assessment scores. Measurements were performed prior to treatment (T0), immediately after repetitive transcranial magnetic stimulation (rTMS) (T1), and 2 months after rTMS (T2). The error bars represent standard deviation for each group. FMA: Fugl-Meyer Assessment; FMA-UE: upper extremity score of FMA; FMA-LE: lower extremity score of FMA; FMA-T: total score of FMA. * *p* < 0.05: comparison with value at T0 in each group.

FMA-UE at T1 and T2 showed a significant improvement compared to that at T0 in each group, respectively (*p* < 0.05). However, FMA-UE showed no significant interaction between time and group (time x group interaction, F_2,2.400_ = 0.040, *p* = 0.961, Figure 3A). The mean improvements in FMA-UE at T1 were 10.6 ± 6.2 and 9.4 ± 11.9, and the mean improvements in FMA-UE at T2 were 15.5 ± 13.4 and 15.5 ± 14.6 in the Cr-Cbll and Cr-sham groups, respectively. There was no significant difference in the mean differences of FMA-UE from T0 to T1 and from T0 to T2 between the Cr-Cbll group and the Cr-sham group (Figure 3D).

FMA-LE at T1 and T2 showed a significant improvement compared to that at T0 in each group, respectively (*p* < 0.05). However, FMA-LE showed no significant interaction between time and group (time x group interaction, F_2,2.017_ = 0.104, *p* = 0.902, Figure 3B). The mean improvements in FMA-LE at T1 were 4.5 ± 3.7 and 5.6 ± 8.5, and the mean improvements in FMA-LE at T2 were 7.5 ± 6.1 and 8.6 ± 8.2 in the Cr-Cbll and Cr-sham groups, respectively. There was no significant difference in the mean differences of FMA-LE from T0 to T1 and from T0 to T2 between the Cr-Cbll group and the Cr-sham group (Figure 3E).

FMA-T at T1 and T2 showed a significant improvement compared to that at T0 in each group, respectively (*p* < 0.05). However, FMA-UE showed no significant interaction between time and group (time x group interaction, F_2,2.217_ = 0.019, *p* = 0.981, Figure 3A). The mean improvements in FMA-T at T1 were 15.1 ± 9.1 and 15.0 ± 17.2, and the mean improvements in FMA-T at T2 were 23.0 ± 18.1 and 24.1 ± 20.2 in the Cr-Cbll and Cr-sham groups, respectively. There was no significant difference in the mean differences of FMA-UE from T0 to T1 and from T0 to T2 between the Cr-Cbll group and the Cr-sham group (Figure 3D).

### 3.2. Changes in Box and Block Test

**Figure 4:**
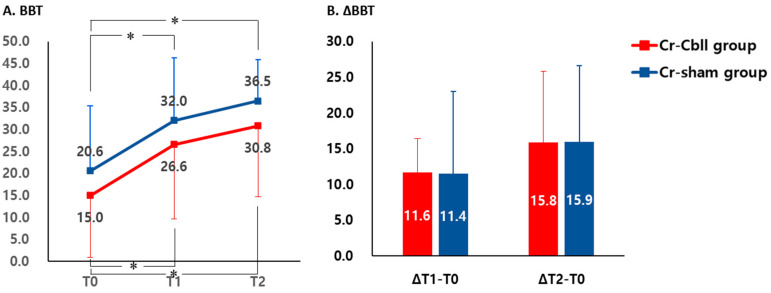
Changes in Box and Block Test. Measurements were performed prior to treatment (T0), immediately after repetitive transcranial magnetic stimulation (rTMS) (T1), and 2 months after rTMS (T2). The error bars represent standard deviation for each group. BBT: Box and Block Test. * *p* < 0.05: comparison with value at T0 in each group.

BBT at T1 and T2 showed a significant improvement compared with that at T0 in each group, respectively (*p* < 0.05). However, BBT did not show a significant interaction between time and group (time x group interaction, F_2,0.117_ = 0.003, *p* = 0.997, Figure 4A). The mean improvements in BBT at T1 were 11.6 ± 4.8 and 11.4 ± 11.6, and the mean improvements in BBT at T2 were 15.8 ± 10.0 and 15.9 ± 10.7 in the Cr-Cbll and Cr-sham groups, respectively. There was no significant difference in the mean differences of BBT from T0 to T1 and from T0 to T2 between the Cr-Cbll group and the Cr-sham group (Figure 4B).

## 4. Discussion

This study showed that sequential facilitatory cerebral and cerebellar rTMS could be a safe rTMS strategy in subacute stroke patients. However, the results of this study could not demonstrate any additional effect of applying the facilitatory rTMS over the contralesional cerebellum in addition to facilitatory rTMS over the affected M1 compared to rTMS over M1 alone, for improving motor function in subacute stroke patients.

The subacute stroke phase is a critical phase for recovery from motor impairment [34]. Therefore, efforts should be made to capture the neuroplasticity changes that occur in the subacute stroke phase to maximize the interaction between spontaneous recovery and training-induced recovery [3]. This implies that the motor learning principle for training-induced recovery and neuroplasticity changes following rTMS should be combined using appropriate timing and methods [4,35,36]. Indeed, appropriate timing of applying rTMS with motor training resulted in better motor recovery than rTMS alone [37]. Therefore, the effect of rTMS on improving motor function in subacute stroke patients may be to enhance the motor learning process.

Based on several previous studies that have implicated the cerebellum in motor learning [20,21,22,23], research on rTMS over the cerebellum has been reported. In previous studies using rTMS in healthy participants, low-frequency rTMS applied to the lateral cerebellum impaired performance on a serial reaction time task [38], and low-frequency rTMS applied over the right paramedian cerebellum impaired performance on a 10-hole pegboard task [39]. These findings are similar to those of fMRI studies, demonstrating that the cerebellum may play an important role in the motor learning process through virtual lesion studies using low-frequency rTMS. In a study with chronic stroke patients, excitatory patterned rTMS over the contralesional cerebellum could promote improvements in gait and balance [17]. These studies with fMRI and rTMS show that the cerebellum may be a potential new target for rTMS to improve motor function in subacute stroke patients, and the hypothesis of the present study was derived from these studies.

However, in this study, there were no significant positive or negative effects of facilitatory rTMS over the cerebellum in addition to facilitatory rTMS over M1 regarding motor recovery. There are several possible explanations for the lack of clarity in this study. This study was conducted in stroke patients with a positive response to TMS-induced MEPs. This means that the stroke patients in this study had a preserved corticospinal tract according to TMS-induced MEPs, suggesting that despite their low initial FMA scores, their prognosis for motor recovery may be favorable [40]. In stroke patients with a mildly affected corticospinal tract, other accessory motor tracts may not have a significant impact on upper extremity motor recovery, and corticospinal tract remediation may be the primary mechanism of motor recovery in stroke patients with preserved corticospinal tract integrity [41,42]. Corticospinal tract remediation may be the primary mechanism of motor recovery in stroke patients with preserved corticospinal tract integrity. Corticocerebellar connections are known to be associated with the prognosis of motor function in stroke patients [43,44,45]. Specifically, the cerebellum and its connections may be involved in promoting motor learning in patients with moderately to severely impaired corticospinal tract integrity after stroke [35]. A recent study reported that the integrity of the dentatothalamocortical tract may be a predictor of upper extremity motor function dependent on the integrity of the cerebrospinal tract [45]. Based on these previous findings, it is likely that motor recovery in subacute stroke patients with a preserved corticospinal tract was most likely due to facilitatory rTMS over M1, which directly stimulates the corticospinal tract, and therefore showed no additional benefit from cerebellar rTMS. To validate this possible explanation, a more detailed quantitative assessment of the extent of damage of the corticospinal tract will be required, including a functional neuroimaging study with a larger number of participants.

Rosso et al. [46] reported that cerebello-motor-paired associative stimulation combined with physiotherapy could improve upper limb motor function in chronic stroke patients. They showed that the conditioning stimulus was delivered over the cerebellum before the test stimulus was delivered over M1. In this study, based on the previous report [24] on sequential neural substrates after motor learning using fMRI, an rTMS protocol was used in which faciliatory stimulation over M1 was delivered first and faciliatory stimulation over the cerebellum was delivered after motor training. Metaplasticity occurs depending on the sequence of rTMS stimulation, which may influence changes in neuroplasticity [47]. Activity-dependent metaplasticity was shown especially when stimulation protocols were administered with a short interval. This supports homeostatic metaplasticity mechanisms in rTMS protocols [47]. Therefore, depending on the sequential rTMS order of M1 and the cerebellum, the pattern of neuroplasticity and motor functional changes may vary depending on the stimulation of each region. Further studies of different stimulation sequences, including the timing of motor training, are needed to clarify this issue.

Many factors are known to influence the recovery of motor function in subacute stroke patients [1]. Among the stroke patients who participated in this study, the two patients who had very mild strokes without sequelae and did not interfere with their daily and social life before the stroke recurrence. In addition, the location of the previous stroke in these two participants was unrelated to motor function. Because this study recruited both ischemic and hemorrhagic stroke patients, it was not possible to compare initial stroke severity, stroke lesion location, and initial treatment characteristics as assessed by the same criteria. This is considered to be one of the limitations of this study. Nevertheless, the influence of other factors on the study results is very small, since there were no differences between the two groups in terms of corticospinal tract integrity assessed by TMS-induced MEPs, baseline FMA score, and age, which have the greatest impact on the effect of rTMS in subacute stroke patients [33]. FMA and Box and Block tests, which were employed in this study, are widely utilized as assessment tools for evaluating motor function in patients with stroke. However, the nature of functional assessment necessitates participant cooperation, which can present a challenge. Obtaining more objective results would be possible if there were a more objective tool that could assess motor function without requiring the participant to cooperate.

This study has some limitations. First, the insufficient number of participants and the relatively high dropout rate were the major limitations of this study. In addition, because this study was not designed with a statistical test for non-equivalence, the equivalence of the two groups cannot be claimed based on the results of this study alone. Further studies with larger numbers of patients are needed to draw more definitive conclusions. Second, inaccuracies in the location of cerebellar rTMS might have contributed. The stimulation site of cerebellar rTMS in this study was determined by the anatomical landmark with the lateral distance from the palpated external protuberance [28]. Anatomical variability in stroke patients may have influenced the results of cerebellar rTMS. If cerebellar rTMS was not applied at the correct location determined by the anatomical landmark, no additional motor improvement could be explained in this study. Future rTMS studies using a neuronavigation system may be needed to verify this possible explanation. In addition, the stroke rehabilitation therapy applied to participants was not strictly standardized. The intensity or amount of rehabilitation therapy applied during the intervention period may have altered the individual motor recovery, although conventional rehabilitation therapy and medication during the intervention period did not differ between the two groups. It is challenging to fully control for conventional rehabilitation therapy, necessitating further studies with larger numbers of participants to reduce the impact.

## 5. Conclusions

This study could not demonstrate the significant additional effect of the cerebellar rTMS over contralesional cerebellum in addition to facilitatory rTMS over affected M1, for improving motor function in subacute stroke patients with TMS-induced MEP response. Although the primary outcome of this study failed to reach statistical significance, its results may serve as indirect evidence for the role of the corticospinal and other accessory motor pathways in motor recovery in subacute stroke patients. It might also be used as a rationale for excluding subacute stroke patients with preserved corticospinal tract as appropriate candidates for a cerebellar rTMS strategy to improve upper extremity motor recovery in the future.

## Figures and Tables

**Table 1:** Baseline demographic and clinical characteristics of the participants (ITT analysis).

Parameters	Cr-Cbll Group (*n* = 10)	Cr-Sham Group (*n* = 10)	*p*-Value
Age (years)	71.6 ± 12.0	66.7 ± 9.8	0.331
Sex (male/female)	7:3	5:5	0.650
Stroke type (ischemic/hemorrhagic)	9:1	8:2	1.000
Stroke recurrence (first ever/recurrent)	9:1	9:1	1.000
Stroke side (right/left)	5:5	6:4	1.000
Stroke lesion (supratentorial/infratentorial)	7:3	7:3	1.000
Stroke duration (days)	15.1 ± 4.2	15.1 ± 6.5	0.316
rMT of affected M1 (%)	59.5 ± 18.6	57.0 ± 16.4	0.753
Fugl-Meyer Assessment			
FMA-UE	37.3 ± 14.2	39.3 ± 16.6	0.776
FMA-LE	17.6 ± 7.3	17.9 ± 7.5	0.929
FMA-T	54.9 ± 18.8	57.2 ± 23.0	0.810
Box and Block Test	15.0 ± 14.1	20.6 ± 14.7	0.396

Values are presented as mean ± SD. *p*-value: comparison between the Cr-Cbll group and the Cr-sham group. rMT: resting motor threshold; M1: primary motor cortex; FMA: Fugl-Meyer Assessment; FMA-UE: upper extremity score of FMA; FMA-LE: lower extremity score of FMA; FMA-T: total score of the FMA.

## Data Availability

The data that support the findings of this study are available from the corresponding author upon reasonable request.
